# On-the-fly resolution enhancement in X-ray protein crystallography using electric field

**DOI:** 10.1007/s00249-025-01731-5

**Published:** 2025-01-22

**Authors:** Krishna Prasad Khakurel, Michal Nemergut, Purbaj Pant, Martin Savko, Jakob Andreasson, Gabriel Žoldák

**Affiliations:** 1 ELI Beamlines Facility, The Extreme Light Infrastructure ERIC, Za Radnicí 835, 25241 Dolní Břežany, Czech Republic; 2 Center for Interdisciplinary Biosciences, Technology and Innovation Park, P. J. Šafárik University, Košice, Slovakia; 3 Soleil Synchrotron, Saint-Aubin, France; 4 Faculty of Sciences, P. J. Šafárik University, Košice, Slovakia

**Keywords:** External electric field, Macromolecular crystals, Resolution enhancement

## Abstract

X-ray crystallography has tremendously served structural biology by routinely providing high-resolution 3D structures of macromolecules. The extent of information encoded in the X-ray crystallography is proportional to which resolution the crystals diffract and the structure can be refined to. Therefore, there is a continuous effort to obtain high-quality crystals, especially for those proteins, which are considered difficult to crystallize into high-quality protein crystals of suitable sizes for X-ray crystallography. Efforts in enhancing the resolution in X-ray crystallography have also been made by optimizing crystallization protocols using external stimuli such as an electric field and magnetic field during the crystallization. Here, we present the feasibility of on-the-fly post-crystallization resolution enhancement of the protein crystal diffraction by applying a high-voltage electric field. The electric field between 2 and 11 kV/cm, which was applied after mounting the crystals in the beamline, resulted in the enhancement of the resolution. The crystal diffraction quality improved progressively with the exposure time. Moreover, we also find that upto defined electric field threshold, the protein structure remains largely unperturbed, a conclusion further supported by molecular dynamics simulations.

**Supplementary Information:**

The online version contains supplementary material available at 10.1007/s00249-025-01731-5.

## Introduction

X-ray crystallography is an established technique, which routinely provides 3D structure of macromolecules (Smyth and Martin 2000). The extent of information in X-ray crystallography is determined by the resolution the structure is refined to Dubach and Guskov (2020), Wlodawer et al. (2008). Therefore, efforts in obtaining high-quality crystal and high-resolution structures are continuously ongoing (Koizumi et al. 2017; Hirano et al. 2016). For some classes of proteins, it is extremely difficult to grow crystals of quality and size suitable for single-crystal X-ray crystallography (Khakurel et al. 2019). Multiple approaches have been adopted in search of enhancing or improving the quality of the crystal and, therefore, the resolution of the obtained structures (Saridakis and Chayen 2000; Hashizume et al. 2020; Guo et al. 2014; McPherson and Cudney 2014). Among them, also include the use of external stimuli such as electric field and magnetic field to enhance the quality of the protein crystals (Alexander and Radacsi 2019; Rubin et al. 2017; Ataka and Wakayama 2002; Ryu et al. 2020).

Resolution in protein crystallography has been a key parameter, which determines the quality of the crystal and the extent of the information encoded (Wlodawer et al. 2008). It also determines how the phase problem can be addressed, and whether the position of light elements and hydrogen can be precisely determined or not (Burla et al. 2003; Elias et al. 2013). Recent efforts in quantum crystallography and ab-initio refinement in X-ray crystallography are also dependent on the resolution to which the crystallographic data can be refined (Bergmann et al. 2020). The success and failure of these approaches are often determined by whether the diffraction of the crystal is beyond a well-defined resolution. Therefore, even a resolution enhancement of the magnitude of sub-angstroms can prove to be beneficial for these methods.

Low-voltage electric field has been used in numerous occasions in the crystallization of the protein crystals (Taleb et al. 1999a; Takashi Wakamatsu and Yuuki Ohnishi 2011). The effect of the electric field on protein crystallization has a rich history with works initiated by Taleb et al. (2001, 1999b) and then contributed by many other researchers (Frontana-Uribe and Moreno 2008; Pareja-Rivera et al. 2017; Flores-Hernández et al. 2013; Sazaki et al. 2004; Koizumi et al. 2013a, 2009; Hammadi and Veesler 2009; Hammadi et al. 2007). Compared to conventional crystallization methods, the use of an electric field in protein crystallization has been reported to produce high-quality crystals (Koizumi et al. 2013b, 2015). However, real-time structural studies in this area remain largely unexplored. Moreover, whether resolution enhancement can be achieved online post-crystallization after mounting the crystals on the beamline has not yet been investigated. This information is particularly critical when crystals diffract but are unsuitable for direct phasing, H-atom positioning, quantum crystallography, or ab-initio refinement. In addition, the search for resolution improvement lacks information on whether applied external stimuli, such as an electric field, change the structure of the protein or not. It is known that an electric field alters the structure of the proteins (Hekstra et al. 2016), but the safe application of the external electric field to increase the resolution without perturbing the structure remains insufficiently explored. While improvement of the crystal quality post-crystallization by chemical methods has been previously discussed (Heras and Martin 2005) achieving similar effects using physical stimuli is largely missing.

Recently, we introduced an in-situ crystallization plate, which allows probing the crystals in the presence of the electric field (Khakurel et al. 2024). The design of the plate allows performing experiments at room temperature and with multiple crystals in the well. Such possibility allows both to explore the structural dynamics of the proteins under the electric field at physiologically more relevant conditions and to explore the gain in the quality of the crystal under the electric field. Here we explore the second possibility and show that the use of high-voltage continuous electric field can be used to enhance the resolution in protein crystallography without introducing significant changes in the structure of the protein.

**Fig. 1:**
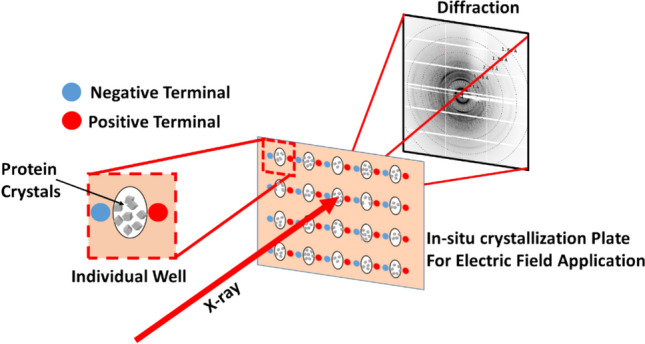
Schematic of on-the-fly application of an external electric field to protein crystals

Furthermore, our approach can also be used to enhance the resolution on-the-fly after determining that the resolution in the absence of the electric field is insufficient for specific purposes, such as ab-initio phasing, H-atom positioning, or ab-initio refinement. As a proof of the principle, lysozyme was used as the model system for our study. A schematic representation of the experimental setup is shown in Fig. 1.

## Materials and methods

### Sample preparation

Lysozyme from chicken egg white (Merck Millipore Ltd., Ireland) was gently dissolved in solubilization buffer (20 mM sodium acetate pH 4.5) at a concentration of ~ 60 mg/mL. Subsequently, the solubilized lysozyme was mixed with the crystallization solution (1.5 M NaCl, 100 mM sodium acetate pH4.5) in a 1:1 ratio. Crystallization was carried out in our designed plate by a batch method in which a maximum of 50 ul of premixed protein and precipitant solutions were sealed and left undisturbed. Lysozyme crystals usually appeared within two days.

### Plate mounting and application of the electric field

The crystallization was done in the 3D-printed in-situ plates, which were equipped with wires acting as electrodes to introduce the electric field. The 3D in-situ plate was mounted to the beamline using the regular plate-screening holder. A custom-designed tunable power supply was used to apply the external electric field. A detailed discussion of the design of the in-situ plate can be found elsewhere (Khakurel et al. 2024). In brief, the 3D-printed frame was designed with nine wells for crystallization, each equipped with the wires acting as the electrodes for the electric field application. The bottom part of the 3D-printed plate was supported by an *in situ* plate seal (MiTeGen, catalogue No. ML-CDSF1-10). Protein solution and crystallization buffer were pipetted into each well, and the plate was then sealed with another *in situ* plate seal. The power supply was assembled using a commercial high-voltage regulated DC-to-DC converter (Ultravolt 30C24-P250-I5), which allows voltage regulation with precision of ~ 0.1%. For the experiments reported in this article, we used an external electric field of 2300 V/cm, 4600 V/cm, 7000 V/cm and 11000 V/cm. We did not proceed with higher voltages due to the limitations of the available power supply and the electrodes.

### Data collection

**Fig. 2:**
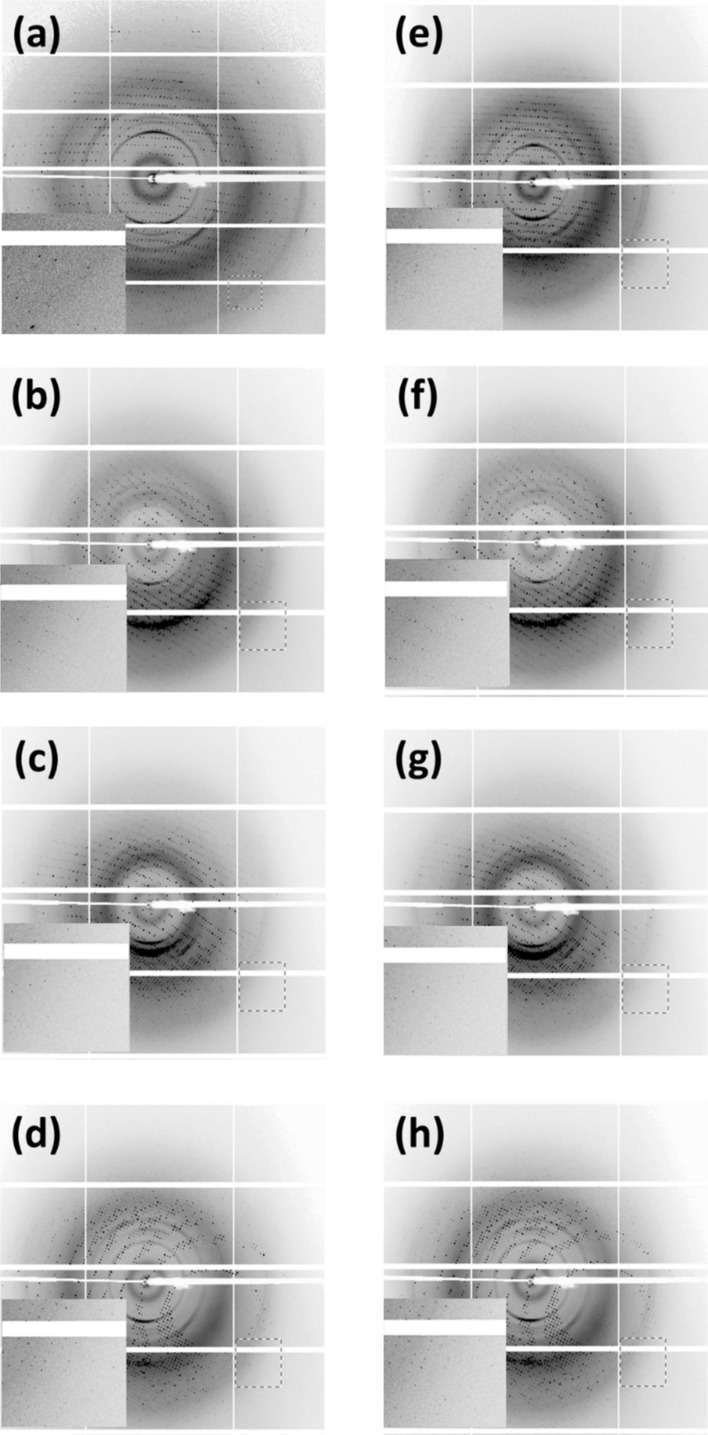
The diffraction pattern of lysozyme collected in the presence and absence of field at different voltages **a** and **e** 2300 V/cm; **b** and **f** 4600 V/cm; **c** and **g** 7000 V/cm; **d** and **h** 11,000 V/cm; The inset shows the diffraction at high resolution indicated by the red box in the main frame

To understand the influence of the high-voltage electric field on the protein crystals and to see if the online enhancement of the crystal diffraction can be realized, we performed an experiment at Proxima 2A crystallography beamline at the SOLEIL synchrotron facility (Duran et al. 2013). For our studies, we used lysozyme crystal as the model system. The in-situ plate that was designed for the electric field application was mounted on the beamline and all the measurements were done at room temperature (295K). The data were collected using 12.65 keV X-rays with a flux of ~ 10^11^ photons/s. The distance between the crystal and the detector was set to ~21.6 cm. All the data presented here have been collected in the angular range of +/− 30 degrees. The oscillation range for a diffraction pattern is 0.1 degrees. For each frame, the exposure time was set to 5 ms. The data were collected for four different electric fields: 2300 V/cm, 4600 V/cm, 7000 V/cm, and 11000 V/cm. The lysozyme crystals used for the data collection were in the range of 20-30 µm. For each dataset, the crystal was translated by ~ 5 µm to ensure that data were collected from a fresh region. For each electric field, the data were collected with and without an electric field and at different time durations the crystals spent in the field. During measurement, for each voltage, we moved to a fresh well with multiple crystals. Figure 2 shows the instances of the diffraction pattern collected with and without electric field for all the electric fields at which the data were collected. In total, data were collected from eight crystals exposed to various electric fields: 3 from 2300 V/cm, 2 from 4600 V/cm, 2 from 7000V/cm, and 1 from 11000 V/cm.

### Data reduction

The data reduction for the dataset collected at different electric fields was done using the software XDS (Kabsch 2010), with which the data were indexed and integrated. The dataset was scaled with AIMLESS (Evans and Murshudov 2013). The resolution was cut-off based on the CC_1/2_ value (Karplus and Diederichs 2015). For all the datasets, the phasing was done with the MolRep (Vagin and Teplyakov 2010) using the lysozyme model with PDB ID:1dpx. The refinement of the structure was done using the Refmac (Murshudov et al. 2011). We performed a similar data analysis for the dataset from the crystal along different time courses at the same electric field. A summary of the data reduction for the different electric fields in the presence and absence of the field is provided in Supplementary Table 1.

### Molecular dynamics simulation

To explore the influence of the high external electric field on the protein crystals, we performed molecular dynamics simulation. For the simulations, we used the same electric fields that were used for the experiments. The electric field simulations are 2300 V/cm, 4600 V/cm, 7000 V/cm, and 11000 V/cm. All the simulations were performed using GROMACS (Hess et al. 2008). All the simulations were carried out using an explicit solvent in the dodecahedron box using the CHARMM-27 force field (Vanommeslaeghe et al. 2010). The protein subjected to simulation was kept at the center of the box such that the distance from the solute to the edge of the box was 10 Å. LINC algorithm (Hess 2008) was used to constrain all the bonds and particle mesh Ewald method (Essmann et al. 1995) with a grid spacing of 10 Å to treat van der Waal interaction. The Coulomb and Lennard-john cut-off was kept as 10 Å. The temperature of all the simulations was kept constant at 300K using a V-rescale thermostat (Bussi et al. 2007) and the constant pressure was controlled at 1 bar using Parrinello—Rahman pressure coupling (Laio and Parrinello 2002). The Tip-3p water model was used as the solvent for the simulation (Price and Brooks 2004). The simulation system was minimized using the steepest descent algorithm and pre-equilibration was performed under NVT and then NPT for 500ps each. A 100 ns production simulation was made where the leapfrog integrator was used with a time step of 2 fs. Each simulated snapshot was saved at an interval of 10 ps for further analysis. For the simulations, the applied electric field was kept constant along the x-direction in the coordinate system used for the simulation.

## Results and discussion

**Fig. 3:**
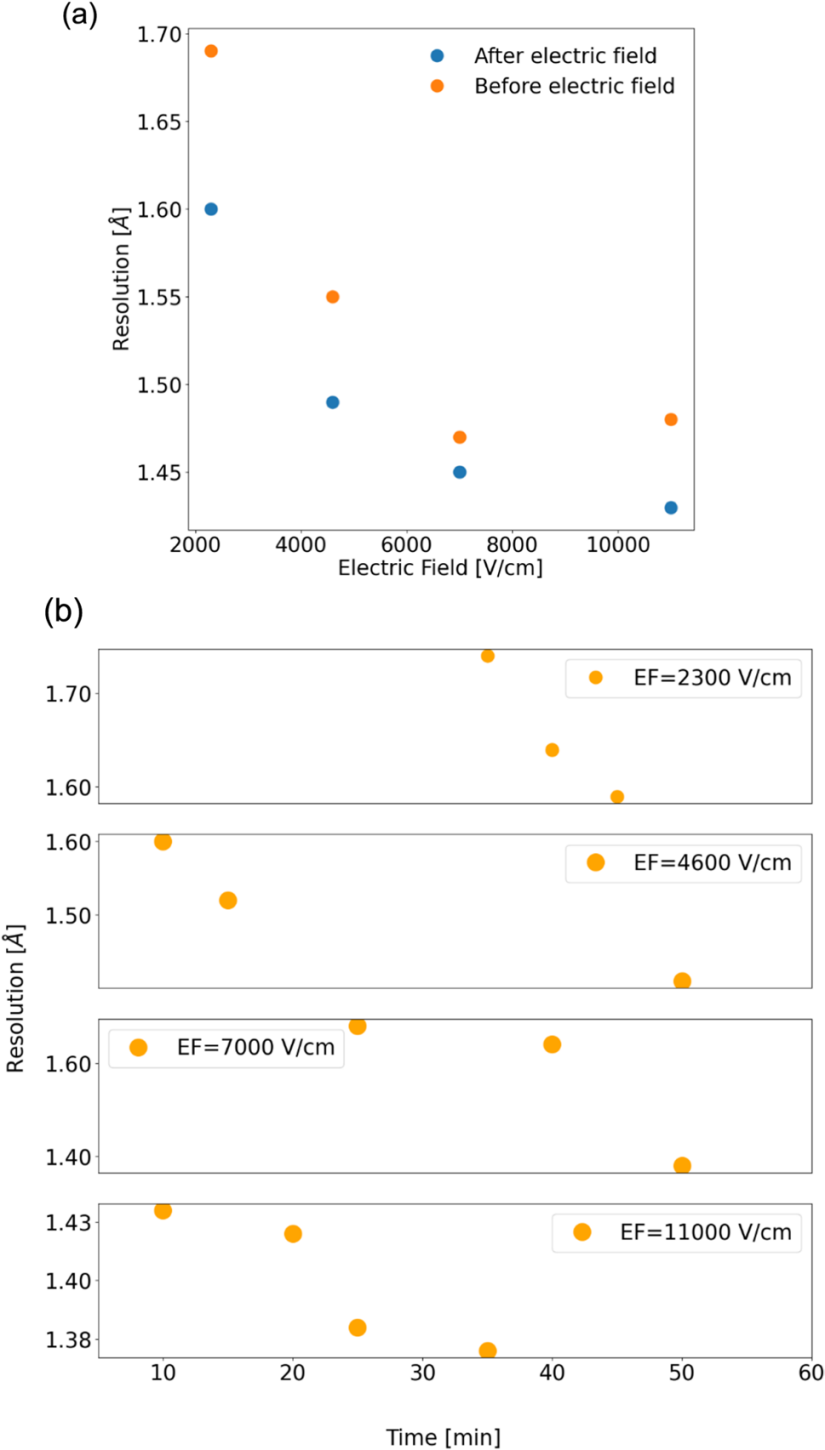
The plot of resolution enhancement in lysozyme crystal: **a** before/after the application of external electric fields; **b** time-dependence of the resolution in different electric fields

The data reduction from the application of the electric field on the crystal structure show that the resolution improves in the presence of the electric field. This is similar to what has been reported when better quality of the crystals is obtained when grown in the presence of the external electric field (Yuan et al. 2022; Rodríguez-Romero et al. 2017). A plot of the resolution obtained from the data reduction is shown in Fig. 3a. For each electric field shown in the figure, a fresh crystal has been used for the measurement of datasets in presence and in absence of the electric field. It can be seen that in the presence of the electric field, the ordering of the crystals improves. We conducted a detailed analysis of the diffraction datasets obtained from crystals exposed to the electric field for varying durations. Our results indicate a clear, progressive improvement in resolution as the exposure time to the electric field increases. This has been observed for all the eight crystals for which such data were collected. A plot of the resolution versus the time the crystal spends in the electric field is shown in Fig. 3b**.** Data for additional crystals are shown in the Supplementary Figure 1, 2 and 3.

**Fig. 4:**
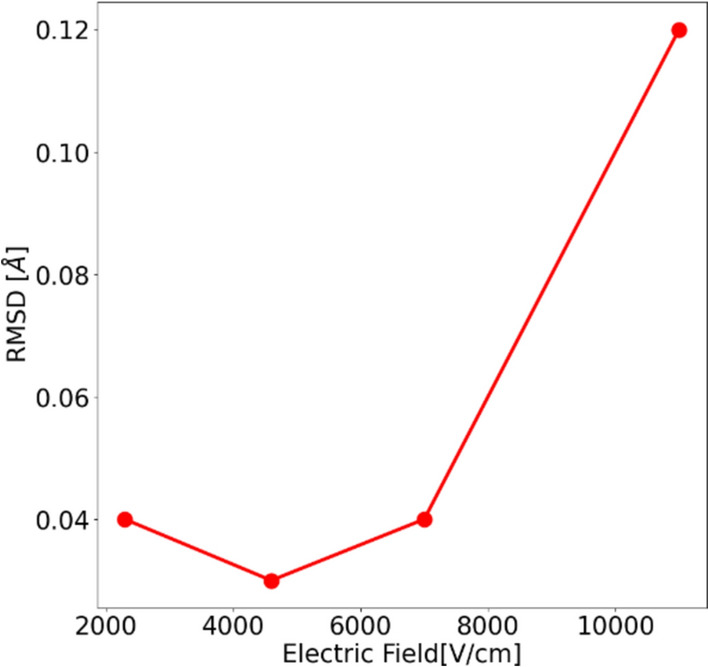
The RMSD of the refined lysozyme structure obtained with and without the field for different applied electric fields

The analysis of the refined electron density maps of the lysozyme in the presence and absence of the electric field showed no change in the densities. We further analyzed the structural models (PDB files) obtained after the refinement in the presence and the absence of the electric field. Root-mean-square deviation (RMSD) serves as a reliable measure to assess whether any changes in the protein structure occurred in response to the external stimuli (Reva et al. 1998). The RMSD between the structure obtained at different electric fields is shown in Fig. 4. In this figure, it can be observed that there is no major change in the RMSD between the models with and without electric fields for electric fields 2300 V/cm, 4600 V/cm, and 7000 V/cm. However, for the electric field of 11000 V/cm, we observe an RMSD of 0.12 Å, which shows an increase compared to the other structures. Yet, the deviation is rather very small and does not introduce significant changes in the structure of the protein. Therefore, we can consider that any voltage below 11000V/cm can be safely used in the resolution (quality) enhancement of the protein crystal.

**Fig. 5:**
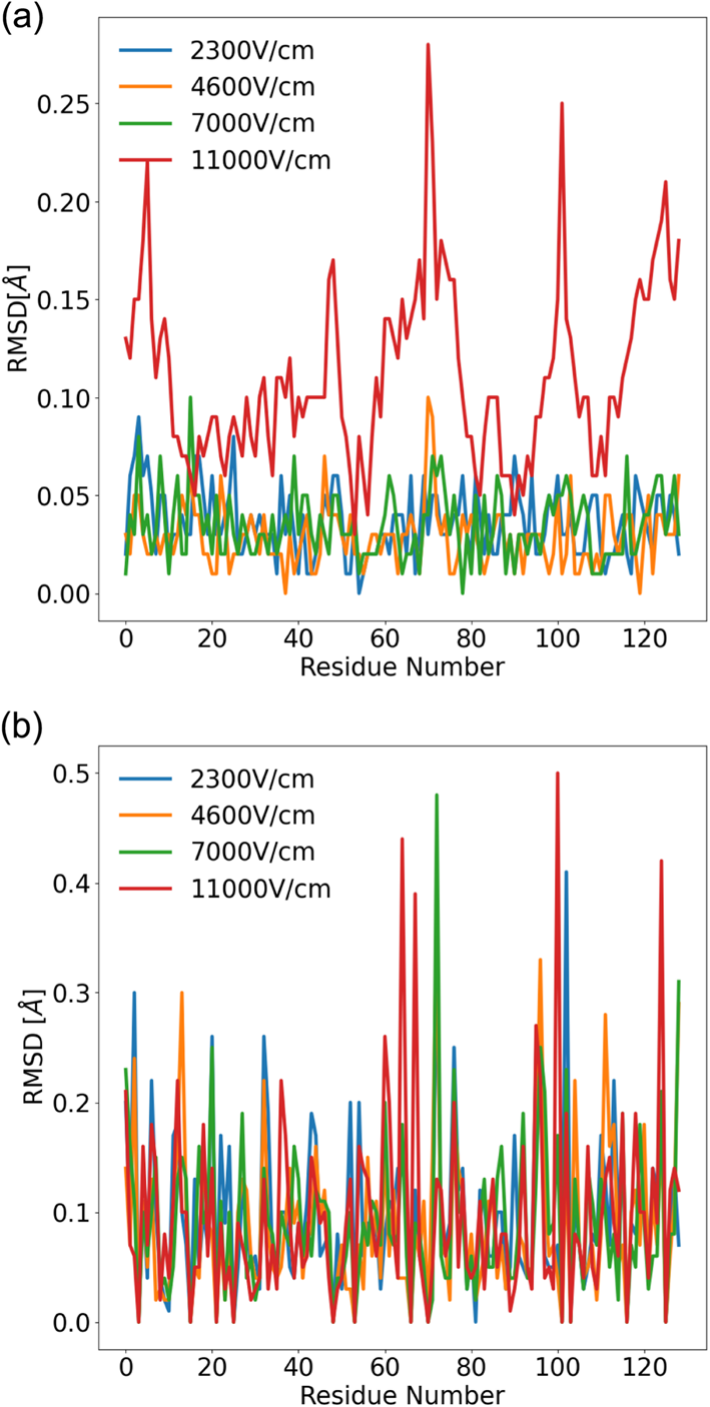
The RMSD of the **a** C-alpha and **b** side chain of the refined lysozyme structure obtained for different electric fields

An analysis of the RMSD of the C-alpha of the structure obtained shows that the RMSD of the residues remains constant for 2300 V/cm, 4600 V/cm, and 7000 V/cm, but the deviation slightly increases for 11000 V/cm. In addiiton the RMSD of the side chain from the structure obtained experimentally at different electric fields confirmed that the changes are minimal and can be considered safe for the usage in the crystal quality enhancement (Fig. 5a and b).

**Fig. 6:**
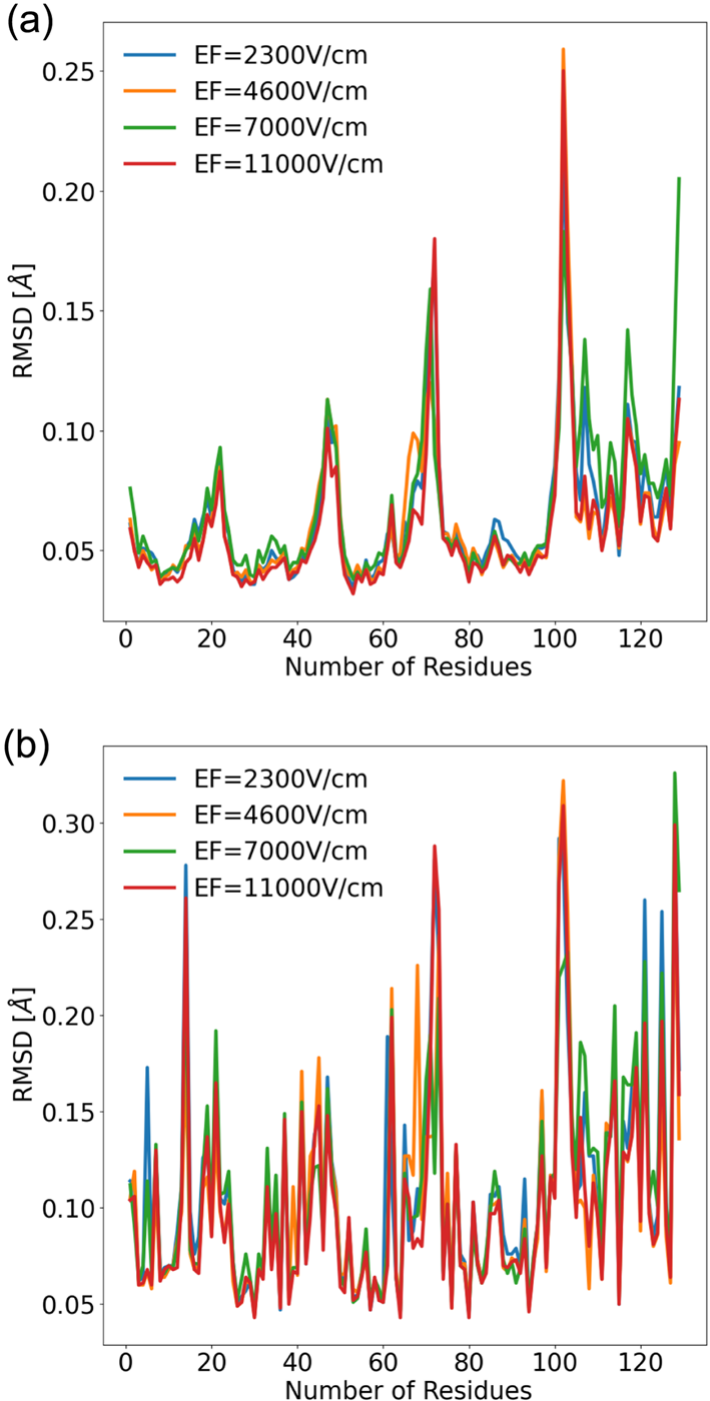
The RMSD of the C-alpha and side chain of lysozyme obtained from MD simulation performed for different applied electric fields

The effect of the external electric field on the C-alpha chain and the side residues as obtained after the post-analysis of the trajectories obtained from the MD simulation is shown in Fig. 6a and b, respectively. The simulation also shows that there are no significant changes in the structure under different fields and aligns with the experimental results that we have obtained.

## Conclusion

To summarize, we have observed the enhancement of the crystal quality in the presence of the electric field. The crystallinity of the protein crystal improves with the time it spends in the electric field. Further, we observed that at up to 7000 V/cm, the structural changes between the electric field on and off state are negligible. The increase in the structural changes starts to appear from the 11,000 V/cm, despite the magnitude being very small. Further experiments are needed to see at which voltage the steep changes start to appear in the structures of the protein. Our results both from the experiment and simulation show that up to 11,000 V/cm, it is safe to use the continuous electric field to enhance the crystal quality post-crystallization. We believe exploring the potentials of the current approach to challenging proteins and in cases where resolutions are critical in phasing; H-atom position will benefit the structural biology community. Our experiment results can also be applied to poorly diffracting crystals, which will be one of the experiments that we shall be exploring in future.

## Supplementary Information

Below is the link to the electronic supplementary material.

Supplementary file1 (DOCX 100 KB)

## Data Availability

Data are available upon request.

## References

[CR1] Alexander LF, Radacsi N (2019) CrystEngComm 21:5014–5031

[CR2] Ataka M, Wakayama NI (2002) Effects of a magnetic field and magnetization force on protein crystal growth. Why does a magnet improve the quality of some crystals? Acta Crystallogr D Biol Crystallogr 58(Pt 10 Pt 1):1708–1710 12351891 10.1107/s0907444902014415

[CR3] Bergmann J, Davidson M, Oksanen E, Ryde U, Jayatilaka D (2020) fragHAR: towards ab initio quantum-crystallographic X-ray structure refinement for polypeptides and proteins. IUCrJ 7(Pt 2):158–165 32148844 10.1107/S2052252519015975 PMC7055371

[CR4] Burla MC, Carrozzini B, Caliandro R, Cascarano GL, De Caro L, Giacovazzo C, Polidori G (2003) Ab initio protein phasing at 1.4 A resolution: the new phasing approach of SIR2003-N. Acta Crystallogr A 59(Pt 6):560–568 14581755 10.1107/s0108767303020257

[CR5] Bussi G, Donadio D, Parrinello M (2007) Canonical sampling through velocity rescaling. J Chem Phys 126(1):014101 17212484 10.1063/1.2408420

[CR6] Dubach VRA, Guskov A (2020) The resolution in X-ray crystallography and single-particle cryogenic electron microscopy. Crystals 10:580

[CR7] Duran D, Couster SL, Desjardins K, Delmotte A, Fox G, Meijers R, Moreno T, Savko M, Shepard W (2013) PROXIMA 2A–a new fully tunable micro-focus beamline for macromolecular crystallography. J Phys Conf Ser 425:012005

[CR8] Elias M, Liebschner D, Koepke J, Lecomte C, Guillot B, Jelsch C, Chabriere E (2013) Hydrogen atoms in protein structures: high-resolution X-ray diffraction structure of the DFPase. BMC Res Notes 2(6):308 10.1186/1756-0500-6-308 PMC3737025 23915572

[CR9] Essmann U, Perera L, Berkowitz ML, Darden T, Lee H, Pedersen LG (1995) A smooth particle mesh Ewald method. J Chem Phys 103(19):8577–8593

[CR10] Evans PR, Murshudov GN (2013) How good are my data and what is the resolution? Acta Crystallogr D Biol Crystallogr 69(Pt 7):1204–1214 23793146 10.1107/S0907444913000061 PMC3689523

[CR11] Flores-Hernández E, Stojanoff V, Arreguín-Espinosa R, Moreno A, Sánchez-Puig N (2013) An electrically assisted device for protein crystallization in a vapor-diffusion setup. J Appl Crystallogr 46:832–834 23682197 10.1107/S0021889813010558 PMC3654317

[CR12] Frontana-Uribe BA, Moreno A (2008) On electrochemically assisted protein crystallization and related methods. Cryst Growth des 8:4194–4199 10.1021/cg301250c PMC3958929 24659923

[CR13] Guo YZ, Sun LH, Oberthuer D et al (2014) Utilisation of adsorption and desorption for simultaneously improving protein crystallisation success rate and crystal quality. Sci Rep 4:7308 25471817 10.1038/srep07308 PMC4255177

[CR14] Hammadi Z, Veesler S (2009) New approaches on crystallization under electric fields. Prog Biophys Mol Biol 101:38–44 20025897 10.1016/j.pbiomolbio.2009.12.005

[CR15] Hammadi Z, Astier JP, Morin R, Veesler S (2007) Protein crystallization induced by a localized voltage. Cryst Growth des 7:1472–1475

[CR16] Hashizume Y, Inaka K, Furubayashi N, Kamo M, Takahashi S, Tanaka H (2020) Methods for obtaining better diffractive protein crystals: from sample evaluation to space crystallization. Crystals 10:78

[CR17] Hekstra D, White K, Socolich M et al (2016) Electric-field-stimulated protein mechanics. Nature 540:400–405 27926732 10.1038/nature20571 PMC5730412

[CR18] Heras B, Martin JL (2005) Post-crystallization treatments for improving diffraction quality of protein crystals. Acta Crystallogr D Biol Crystallogr 61(Pt 9):1173–1180 16131749 10.1107/S0907444905019451

[CR19] Hess B (2008) P-LINCS: a parallel linear constraint solver for molecular simulation. J Chem Theory Comput 4(1):116–122 26619985 10.1021/ct700200b

[CR20] Hess B et al (2008) Gromacs 4: algorithms for highly efficient, load-balanced, and scalable molecular simulation. J Chem Theor Comput 4:435–447 10.1021/ct700301q 26620784

[CR21] Hirano Y, Takeda K, Miki K (2016) Charge-density analysis of an iron–sulfur protein at an ultra-high resolution of 0.48 Å. Nature 534:281–284 27279229 10.1038/nature18001

[CR22] Kabsch W (2010) XDS. Acta Cryst D66:125–132 10.1107/S0907444909047337 PMC2815665 20124692

[CR23] Karplus PA, Diederichs K (2015) Assessing and maximizing data quality in macromolecular crystallography. Curr Opin Struct Biol 34:60–68 26209821 10.1016/j.sbi.2015.07.003 PMC4684713

[CR24] Khakurel KP, Angelov B, Andreasson J (2019) Macromolecular Nanocrystal structural analysis with electron and X-rays: a comparative review. Molecules 24:3490 31561479 10.3390/molecules24193490 PMC6804143

[CR25] Khakurel KP, Nemergut M, Džupponová V, Kropielnicki K, Savko M, Žoldák G, Andreasson J (2024) Design and fabrication of 3D-printed in situ crystallization plates for probing microcrystals in an external electric field. J Appl Crystallogr 57(Pt 3):842–847 38846773 10.1107/S1600576724002140 PMC11151662

[CR26] Koizumi H, Fujiwara K, Uda S (2009) Control of nucleation rate for tetragonal hen-egg white lysozyme crystals by application of an electric field with variable frequencies. Cryst Growth des 9:2420–2424

[CR27] Koizumi H, Uda S, Fujiwara K, Tachibana M, Kojima K, Nozawa J (2013a) Improvement of crystal quality for tetragonal hen egg white lysozyme crystals under application of an external alternating current electric field. J Appl Crystallogr 46:25–29

[CR28] Koizumi H, Uda S, Fujiwara K, Tachibana M, Kojima K, Nozawa J (2013b) Improvement of crystal quality for tetragonal hen egg white lysozyme crystals under application of an external alternating current electric field. J Appl Crystallogr 46(1):25–29

[CR29] Koizumi H, Uda S, Fujiwara K, Tachibana M, Kojima K, Nozawa J (2015) Crystallization of high-quality protein crystals using an external electric field. J Appl Crystallogr 48(5):1507–1513

[CR30] Koizumi H, Uda S, Tsukamoto K, Tachibana M, Kojima K, Okada J, Nozawa J (2017) Crystallization technique of high-quality protein crystals controlling surface free energy. Cryst Growth des 17(12):6712–6718

[CR31] Laio A, Parrinello M (2002) Escaping free-energy minima. Proc Natl Acad Sci U S A 99(20):12562–12566 12271136 10.1073/pnas.202427399 PMC130499

[CR32] McPherson A, Cudney B (2014) Optimization of crystallization conditions for biological macromolecules. Acta Crystallogr F Struct Biol Commun 70(Pt 11):1445–1467 25372810 10.1107/S2053230X14019670 PMC4231845

[CR33] Murshudov GN, Skubák P, Lebedev AA, Pannu NS, Steiner RA, Nicholls RA, Winn MD, Long F, Vagin AA (2011) REFMAC5 for the refinement of macromolecular crystal structures. Acta Crystallogr D Biol Crystallogr 67(Pt 4):355–367 21460454 10.1107/S0907444911001314 PMC3069751

[CR34] Pareja-Rivera C, Cuéllar-Cruz M, Esturau-Escofet N, Demitri N, Polentarutti M, Stojanoff V, Moreno A (2017) Recent advances in the understanding of the influence of electric and magnetic fields on protein crystal growth. Cryst Growth des 17:135–145

[CR35] Price DJ, Brooks CL III (2004) A modified TIP3P water potential for simulation with Ewald summation. J Chem Phys 121(20):10096–10103 15549884 10.1063/1.1808117

[CR36] Reva BA, Finkelstein AV, Skolnick J (1998) What is the probability of a chance prediction of a protein structure with an rmsd of 6 A? Fold des 3(2):141–147 9565758 10.1016/s1359-0278(98)00019-4

[CR37] Rodríguez-Romero A, Esturau-Escofet N, Pareja-Rivera C, Moreno A (2017) Crystal growth of high-quality protein crystals under the presence of an alternant electric field in pulse-wave mode, and a strong magnetic field with radio frequency pulses characterized by X-ray diffraction. Crystals 7(6):179

[CR38] Rubin E, Owen C, Stojanoff V (2017) Crystallization under an external electric field: a case study of glucose isomerase. Crystals 7:206

[CR39] Ryu SY, Oh IH, Cho SJ, Kim SA, Song HK (2020) Enhancing protein crystallization under a magnetic field. Crystals 10:821

[CR40] Saridakis E, Chayen NE (2000) Improving protein crystal quality by decoupling nucleation and growth in vapor diffusion. Protein Sci 9(4):755–757 10794418 10.1110/ps.9.4.755 PMC2144611

[CR41] Sazaki G, Moreno A, Nakajima K (2004) Novel coupling effects of the magnetic and electric fields on protein crystallization. J Cryst Growth 262:499–502

[CR42] Smyth MS, Martin JH (2000) X-ray crystallography. Mol Pathol 53(1):8–14 10884915 10.1136/mp.53.1.8 PMC1186895

[CR43] Taleb M, Didierjean C, Jelsch C, Mangeot J, Capelle B, Aubry A (1999a) Crystallization of proteins under an external electric field. J Crystal Growth 200(3):575–582

[CR44] Taleb M, Didierjean C, Jelsch C, Mangeot JP, Capelle B, Aubry A (1999b) Crystallization of proteins under an external electric field. J Cryst Growth 200:575–582

[CR45] Taleb M, Didierjean C, Jelsch C, Mangeot JP, Aubry A (2001) Equilibrium kinetics of lysozyme crystallization under an external electric field. J Cryst Growth 232:250–255

[CR46] Vagin A, Teplyakov A (2010) Molecular replacement with MOLREP. Acta Crystallogr D Biol Crystallogr 66(Pt 1):22–25 20057045 10.1107/S0907444909042589

[CR47] Vanommeslaeghe K, Hatcher E, Acharya C, Kundu S, Zhong S, Shim J, Darian E, Guvench O, Lopes P, Vorobyov I, Mackerell AD Jr (2010) CHARMM general force field: a force field for drug-like molecules compatible with the CHARMM all-atom additive biological force fields. J Comput Chem 31(4):671–690 19575467 10.1002/jcc.21367 PMC2888302

[CR48] Wakamatsu T, Ohnishi Y (2011) Transparent cell for protein crystallization under low applied voltage. Jpn J Appl Phys 50(4R):048003

[CR49] Wlodawer A, Minor W, Dauter Z, Jaskolski M (2008) Protein crystallography for non-crystallographers, or how to get the best (but not more) from published macromolecular structures. FEBS J 275(1):1–21 18034855 10.1111/j.1742-4658.2007.06178.x PMC4465431

[CR50] Yuan Z, Wu M, Meng Y, Niu Y, Xiao W, Ruan X, He G, Jiang X (2022) Protein crystal regulation and harvest via electric field-based method. Curr Opin Chem Eng 36:100744

