# Supplementary material for: On-the-fly resolution enhancement in X-ray protein crystallography using electric field
Source: Eur Biophys J. 2025 Jan 22;54(1-2):89–95. doi: 10.1007/s00249-025-01731-5 (PMC11880155; doi:10.1007/s00249-025-01731-5)

**On-the-fly resolution enhancement in X-ray protein crystallography using electric field**

Krishna Prasad Khakurela*, Michal Nemergut,^b^ Purbaj Pant,^a^ Martin Savko,^c^ Jakob Andreasson^a^ and Gabriel Žoldák^d^

aELI Beamlines Facility, The Extreme Light Infrastructure ERIC, Za Radnicí 835, 25241 Dolní Břežany

bCenter for Interdisciplinary Biosciences, Technology and Innovation Park, P. J. Šafárik University, Košice, Slovakia,

cSoleil Synchrotron, Saint-Aubin, France

^d^Faculty of Sciences, P. J. Šafárik University, Košice, Slovakia,

*Corresponding author: Krishna.Khakurel@eli-beams.eu

**Table 1** A summary of the data reduction of the data obtained in the absence and presence of the electric field.

| Data Collection | 2300 V/cm | | 4600 V/cm | | 7000 V/cm | | 11000 V/cm | |
| --- | --- | --- | --- | --- | --- | --- | --- | --- |
|  | Without Field | With Field | Without Field | With Field | Without Field | With Field | Without Field | With Field |
| Wavelength (Å) | 0.98 | 0.98 | 0.98 | 0.98 | 0.98 | 0.98 | 0.98 | 0.98 |
| Space group | *P*4_3_2_1_2 | *P*4_3_2_1_2 | *P*4_3_2_1_2 | *P*4_3_2_1_2 | *P*4_3_2_1_2 | *P*4_3_2_1_2 | *P*4_3_2_1_2 | *P*4_3_2_1_2 |
| Cell dimensions |  |  |  |  |  |  |  |  |
| a, b, c (Å) | 79.14, 79.14, 37.88 | 79.14, 79.14, 37.88 | 79.14, 79.14, 37.88 | 79.14, 79.14, 37.88 | 79.14, 79.14, 37.88 | 79.14, 79.14, 37.88 | 79.14, 79.14, 37.88 | 79.14, 79.14, 37.88 |
| α, β, γ (°) | 90, 90, 90 | 90, 90, 90 | 90, 90, 90 | 90, 90, 90 | 90, 90, 90 | 90, 90, 90 | 90, 90, 90 | 90, 90, 90 |
| Resolution (Å) | 1.69  (1.75-1.69) | 1.60  (1.65-1.60) | 1.55  (1.60-1.55) | 1.49  (1.55-1.49) | 1.47 (1.5-1.47) | 1.45  (1.51-1.45) | 1.48 (1.51-1.48) | 1.44  (1.51-1.43) |
| Total reflections | 38067  (261) | 38911 (253) | 65205  (2581) | 65345  (2527) | 82223  (3366) | 81132  (3320) | 83472  (3478) | 81132  (3320) |
| Unique reflections | 12463  (160) | 14098  (196) | 16742  (721) | 16501  (692) | 19347  (969) | 18665  (933) | 19108 (973) | 18665  (933) |
| Rmerge | 0.057  (0.735) | 0.059 (0.443) | 0.095 (0.758) | 0.121 (0.640) | 0.153  (1.106) | 0.144  (0.734) | 0.168  (1.05) | 0.144  (0.734) |
| I/σI | 10.0  (0.9) | 8 (1.8) | 8.8 (1.7) | 8.5 (2.0) | 8.8  (1.6) | 8.5  (1.4) | 5.5  (1.0) | 8.5  (1.4) |
| Multiplicity | 3.1  (1.6) | 3.3(3.1) | 4.4 (4.6) | 4.0(3.7) | 4.2(3.5) | 4.3 (3.6) | 4.4 (3.6) | 4.3 (3.6) |
| Completeness (%) | 84.7  (23.2) | 95.6 (98.4) | 93.1 (82.1) | 92.0 (79.2) | 92.2 (94.4) | 90.3 (94.2) | 90.2 (95.0) | 90.3 (94.2) |


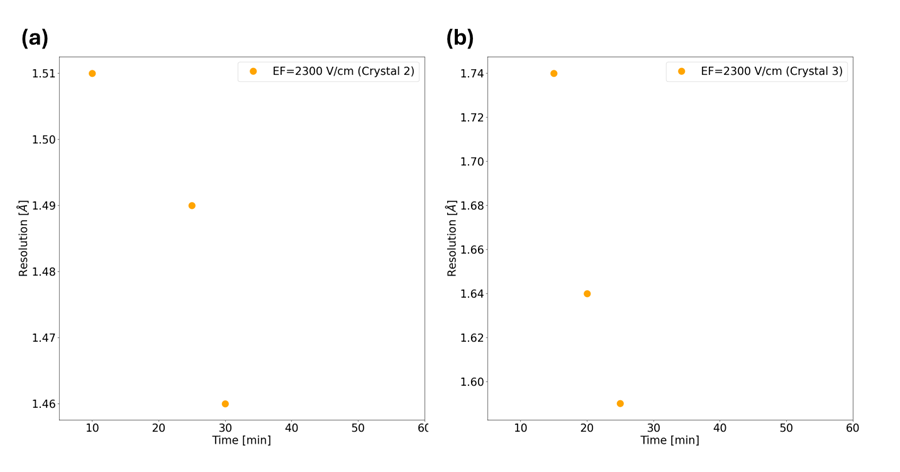
Suppl. Fig.1 Resolution enhancement of crystals under electric field 2300 V/cm (a) for second crystal and (b) for third crystal

Suppl. Fig.2 Resolution enhancement of crystals under electric field 4600 V/cm for second crystal


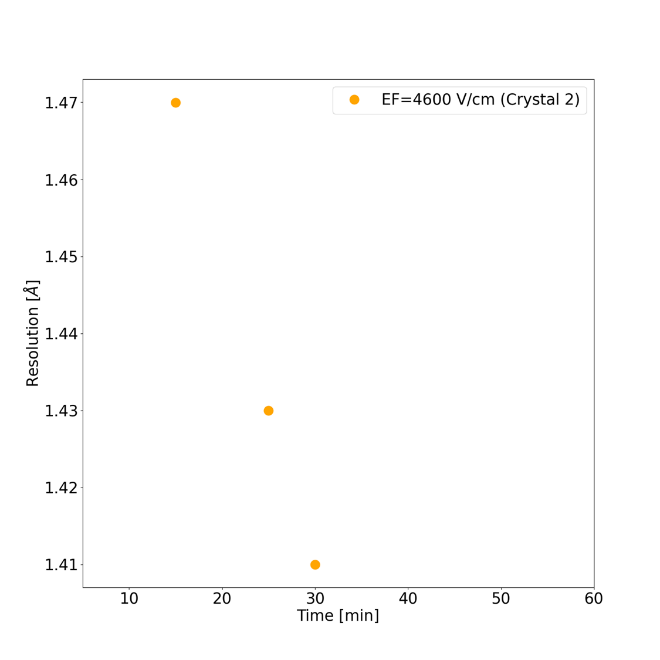


Suppl. Fig.3 Resolution enhancement of crystals under electric field 7000 V/cm for second crystal


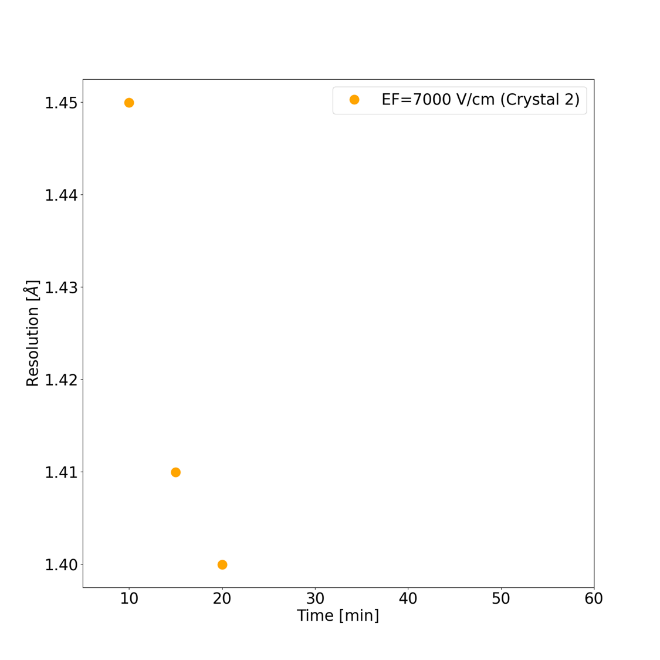

Supplement: Supplementary file 1 — Supplementary file1 (DOCX 100 KB) [file 249_2025_1731_MOESM1_ESM.docx]
